# Exploring the nature of stuttering through a behavioral-neuro-modulation intervention program in bilinguals with stuttering

**DOI:** 10.1590/2317-1782/e20240186en

**Published:** 2025-01-27

**Authors:** Chanchal Chaudhary, Samir Kumar Praharaj, Gopee Krishnan

**Affiliations:** 1 Department of Speech and Hearing, Manipal College of Health Professions, Manipal Academy of Higher Education, Manipal, Karnataka, India.; 2 Department of Audiology and Speech-Language Pathology, Kasturba Medical College, Mangalore, Manipal Academy of Higher Education, Manipal, Karnataka, India.; 3 Department of Psychiatry, Kasturba Medical College, Manipal Academy of Higher Education, Manipal, Karnataka, India.

**Keywords:** Stuttering, Transcranial Direct Current Stimulation, Bilingualism, Childhood-onset Fluency Disorder, Intervention

## Abstract

**Purpose:**

Investigations on identifying the nature of stuttering present varying views. The argument remains whether the stuttering dysfluencies have a motor or a linguistic foundation. Though stuttering is considered a speech-motor disorder, linguistic factors are increasingly reported to play a role in stuttering. Current literature points towards deficits in speech-related motor areas of the brain to abnormalities in linguistic planning and phonological memory playing a role in stuttering. Examining cross-linguistic generalization of treatment gains from treated to untreated language in bilinguals who stutter may provide a unique opportunity to explore the motor and linguistic factors in stuttering.

**Methods:**

In the current study, we explored this potential by experimentally controlling the language of treatment in bilinguals with stuttering (BWS). We hypothesized that if the dysfluencies in stuttering arise from the underlying motor deficits, then the language of treatment would not play a significant role in cross-linguistic generalization. Sixteen BWS were given transcranial direct current stimulation (tDCS) along with behavioral intervention for three weeks. The language of treatment was randomized, wherein participants in one group received behavioral intervention in their dominant language and the other in their non-dominant language.

**Results:**

Results showed that participants in both groups showed a reduction in their stuttering dysfluencies (% stuttered syllables) regardless of the language of treatment, and the treatment gains were generalized to the non-treated language.

**Conclusion:**

Linguistic factors such as language dominance and structure of languages did not surface to play a role in the generalization, signaling the motoric nature of dysfluencies in stuttering.

## INTRODUCTION

Decades of research employing behavioral and neuroimaging paradigms on the nature of dysfluencies in stuttering hover around motor versus linguistic substrates of this speech disorder^(1)^. Literature on the neural correlates of stuttering shows subtle structural and functional anomalies in the speech-related motor areas in the brains of persons who stutter (PWS) as compared to people with no stuttering (PWNS)^(2-7)^. Reduced activations in the left speech motor areas and increased activations in the contralateral right regions are frequently observed in PWS^(3-5)^. Other areas that show reduced activity include the left supplementary motor area (SMA) and inferior frontal regions^(3,6,7)^. These findings collectively point toward deficits in motor response initiation and timely activation in speech-related motor areas, leading to dysfluencies in stuttering.

On the other hand, several linguistic factors are reported to play a role in stuttering^(8)^. The association between the occurrence of stuttering and linguistic planning/ execution is indicative of linguistic deficits in stuttering^(9)^ and neural abnormalities associated with phonological working memory deficits^(10)^. Poor performance in retaining and reproducing novel phonological sequences is interpreted as the linguistic nature of stuttering^(11)^. Corroboratively, neuroimaging data show evidence of abnormalities in both language and speech areas in the brain^(1)^. Watkins et al.^(1)^ argued that though stuttering is expressed in its motor characteristics, the cause might not be purely in the motor system as certain linguistic factors impact stuttering frequency.

Investigations on cross-linguistic generalization of treatment effects in bilinguals who stutter (BWS) highlight the factors underlying the nature of stuttering^(12,13)^. These include both language-related and motoric factors. Language-related factors include language dominance, proficiency^(13)^, and structural similarity between languages. Motor factors include the practice effect observed in the untrained language following training in another language^(12)^. Delving deep into these factors may shed light on the real nature (i.e., motor vs linguistic) of stuttering.

Lim et al.^(13)^ used a speech restructuring program on 19 BWS in English, and the outcomes were measured in both English and Mandarin spoken by them. Stuttering reduction in English was generalized to Mandarin. The authors suggested that the altered speech rate in English was automatically transferred to the untreated Mandarin language. However, how these treatment effects were transferred was not scientifically investigated in their study. Vong et al.^(14)^ reported similar findings from a single-subject study on four Malaysian bilingual children using the Lidcombe program. In two children who underwent the treatment program, stuttering was reduced in both treated and untreated languages, showing positive treatment outcomes. Among the other two participants, one did not reach stage 2 of the program, and for the other, the post-treatment data were unavailable to make a comparison. In another study^(12)^ on a cross-linguistic generalization of treatment (non-programmed prolonged speech technique), five BWS who received treatment in their first (and preferred) language showed improvement in both their treated and untreated languages (i.e., generalization of treatment gains).

A common observation from these studies is that the language of treatment was not controlled or decided based on any scientific reason. Rather, it was merely selected based on participants’ or clinician’s preferences. Further, the participants were not given explicit instructions to avoid using the techniques in the untreated language. However, in previous interventional studies in BWS that altered the speech characteristics, there was no way to document the use of learned techniques in the untreated language. This limitation made the authors’ postulations on the mechanism (linguistic versus motoric) behind the generalization of treatment effects to the untrained language speculative.

In the current study, we maintained that the mechanism underlying stuttering (i.e., linguistic versus motor) can be determined by the experimental manipulation of these factors rather than merely observing and comparing the dysfluencies in two languages^(15-18)^. Thus, the current study aimed to investigate the nature of stuttering by experimentally controlling the language of treatment in BWS.

## METHOD

### Ethics statements

The study was initiated after obtaining approval from the institutional ethics committee (IEC:900/2018). All recruited participants read the participant information sheet and signed the written informed consent form before being part of the study.

### Study design

A parallel-group randomized trial was conducted in which the participants were divided into two groups. Group A received tDCS stimulation along with behavioral intervention in their dominant language, while Group B received the same stimulation and behavioral intervention in their non-dominant language.

### Participants

**Figure 1:**
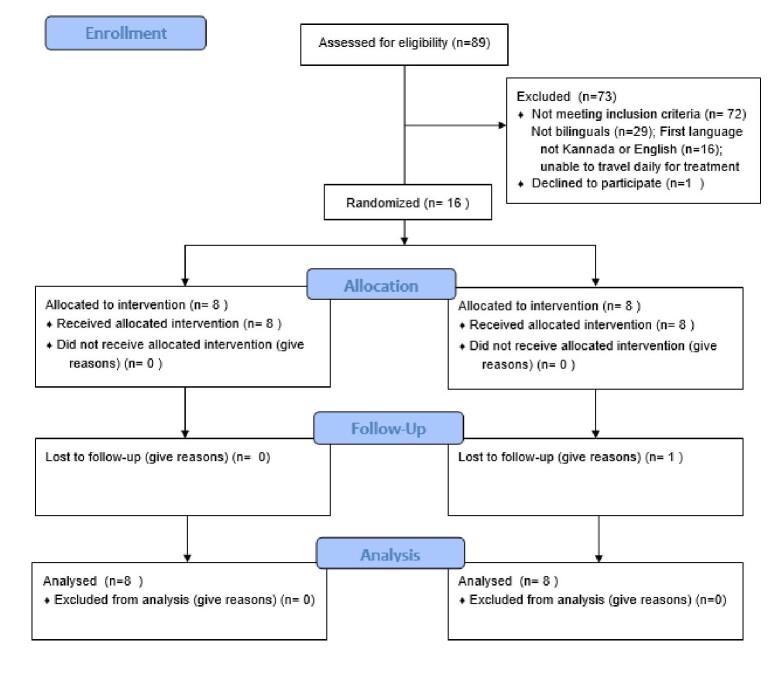
CONSORT Flowchart

A total of 89 participants who visited the outpatient department of a tertiary care hospital with complaints of stuttering were screened for eligibility. Of these, 16 Kannada-English bilinguals were recruited for the study, while others were excluded (see Figure 1 for the reasons for exclusion). The recruited participants had a mean age of 21.13 years (SD=2.90; range: 18-35 years). To qualify for participation in this study, all were diagnosed with stuttering using Stuttering Severity Instrument-4 (SSI-4)^(19)^. Additionally, they either had Kannada or English as their first or second language and were classified as either ‘Kannada-dominant’ or ‘English-dominant’ based on a self-report classification tool (Lim et al.^(16)^), adapted and validated for Kannada-English bilinguals. Finally, all participants were exposed to both languages before age seven. The participants ' demographic details and language characteristics are provided in Tables 1 and 2, respectively.

**Table 1:** Participant details

Participant code	Age	Occupation	Dominant Language	Treatment group^a^	Language of treatment
XX5	19	Student	English	B	Kannada
KT4	19	Student	Kannada	A	Kannada
NY1	23	Software Engineer	Kannada	B	English
BR0	22	Civil Engineer	Kannada	A	Kannada
UL6	21	Student	Kannada	B	English
MI1	18	Student	Kannada	B	English
NI3	21	Engineer	English	A	English
JR8	18	Student	English	A	English
GP6	19	Student	Kannada	A	Kannada
XV7	20	Nurse	Kannada	B	English
QM4	23	Builder	Kannada	A	Kannada
GH9	23	Auditor	Kannada	B	English
TH7	21	Student	Kannada	B	English
WT4	22	Student	Kannada	B	Kannada
SN1	20	Student	English	A	English
NB1	27	Engineer	Kannada	A	Kannada

a Participants in Group A were treated in their dominant language and participants in group B were treated in their non-dominant language

**Table 2:** Language Characteristics of Participants

	**Group A**	**Group B**	**p value** *
	**(n=8)**	**(n=8)**	
Age (mean, SD)	21.12 (2.90)	20.87 (1.80)	0.84
Age of exposure to Kannada	1.28 (1.75)	0.75 (1.16)	0.49
Age of exposure to English	5 (1.41)	5.37 (1.06)	0.57
Years of exposure to Kannada	18.26 (2.43)	18.75 (2.31)	0.69
Years of exposure to English	16 (2.56)	16.75 (3.73)	0.65

* p<0.05

**Caption:** SD, Standard Deviation,

### Randomization

A research scholar who was not a part of the study generated the randomization list using a computer random generator. We used the block randomization method. The same investigator prepared the randomization sequence using sequentially numbered opaque, sealed envelopes. The allocation sequence remained concealed till the assignment. Figure 1 (CONSORT Flowchart) depicts the details of the recruitment, randomization, and allocation of participants to the two groups.

### Intervention details

**Figure 2:**
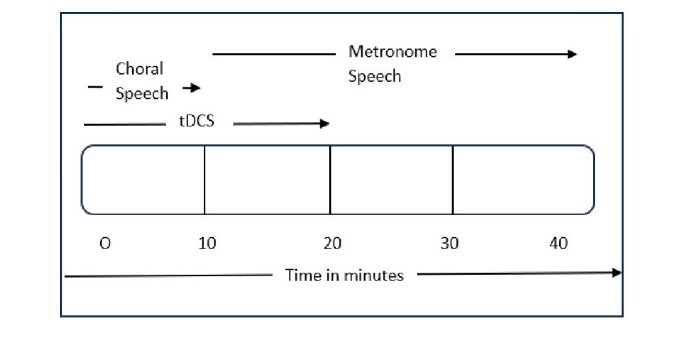
Intervention timeline

As mentioned in the Study Design, Group A participants received tDCS along with a behavioral intervention in their dominant language. In contrast, Group B participants received tDCS along with behavioral intervention in their non-dominant language. Each participant underwent treatment for three weeks (5 days per week), with each session lasting 40 minutes. During the first half of the session, tDCS was administered along with behavioral intervention, after which the stimulator was turned off, and the behavioral intervention continued for the rest of the session. This was done to maximize the benefits of stimulation, as increased excitability can last up to several hours post-stimulation^(20)^. The flow of intervention delivery is depicted in Figure 2.

### Procedure

In this study, we followed the treatment paradigm used by Chesters et al.^(21)^. The site of stimulation was area FC5 (left inferior frontal cortex) on the 10-10 electrode system for EEG recording. This area has consistently shown atypical activation patterns in individuals who stutter^(3)^, and studies have shown a reduction in stuttering dysfluencies following tDCS stimulation of this area^(21,22)^. The anode was placed over area FC5, while the cathode was placed over the right supraorbital ridge. The current stimulation was delivered at 1 mA current for 20 minutes, after which the stimulator was turned off. The participants were instructed to report any discomfort experienced during the stimulation.

For behavioral intervention, we used choral speech followed by a metronome speech task. In the choral speech task, audio samples in both English and Kannada languages were prerecorded using standardized passages and sentences. In the metronome task, participants were instructed to speak along with an external metronome beat at a rate comfortable to the participants^(21)^.

### Outcome measures

We recorded speech samples at baseline and at the end of each week during the four-week intervention period, i.e., at baseline, end of 5th day, end of 10th day, and end of 15th day of intervention (post-intervention). The samples collected at each time-point included reading, conversation, and monologue tasks in Kannada and English. Subsequently, we transcribed these samples and calculated the percentage of stuttered syllables (%SS), which was the primary outcome measure. No formal tool was used to document the tDCS adverse effects; however, we instructed the participants to report any discomfort or side effects experienced during the intervention.

### Statistical analysis

The primary outcome measure, the percentage of stuttered syllables %SS, was expressed using descriptive statistics (Table 3). For data analysis, we used the Linear Mixed Effects method (implemented in Jamovi, version: 2.3.21) with participants as the Random effect and Group and Timepoint as the Fixed effects.

**Table 3:** Percentage of stuttered syllables (%SS) at baseline and post-intervention in both the languages (Kannada and English)

Group	Timepoint	Language	Mean	SD	Median	IQR
A	0	Kannada	8.85	4.62	7.87	6.11-11.9
		English	11.1	4.54	10.6	8.63-14.7
	15	Kannada	3.05	2.29	2.14	1.43-4.52
		English	4.79	3.89	3.14	1.57-7.92
B	0	Kannada	6.22	3.25	5.04	4.21-7.63
		English	7.76	5.73	5.92	4.08-8.50
	15	Kannada	1.99	1.61	1.52	1.20-2.28
		English	2.4	1.68	2.00	1.47-3.20

**Caption:** SD, Standard Deviation, IQR, Interquartile range

## RESULTS

At baseline, participants in both groups had similar characteristics. The mean age of participants was 21.12 (2.90) years in group A and 20.87 (1.80) years in group B. Independent samples t-test showed no significant difference in baseline characteristics between the groups in terms of their age (p=0.84) and language characteristics, such as the age of exposure and the number of years exposed to Kannada and English languages (Table 2). Table 3 provides the descriptive statistics for %SS as a function of Group, Timepoint, and Language. Upon visual inspection of the data, %SS showed a reduction for participants in both groups and their dominant and non-dominant languages (i.e., English or Kannada).

**Figure 3:**
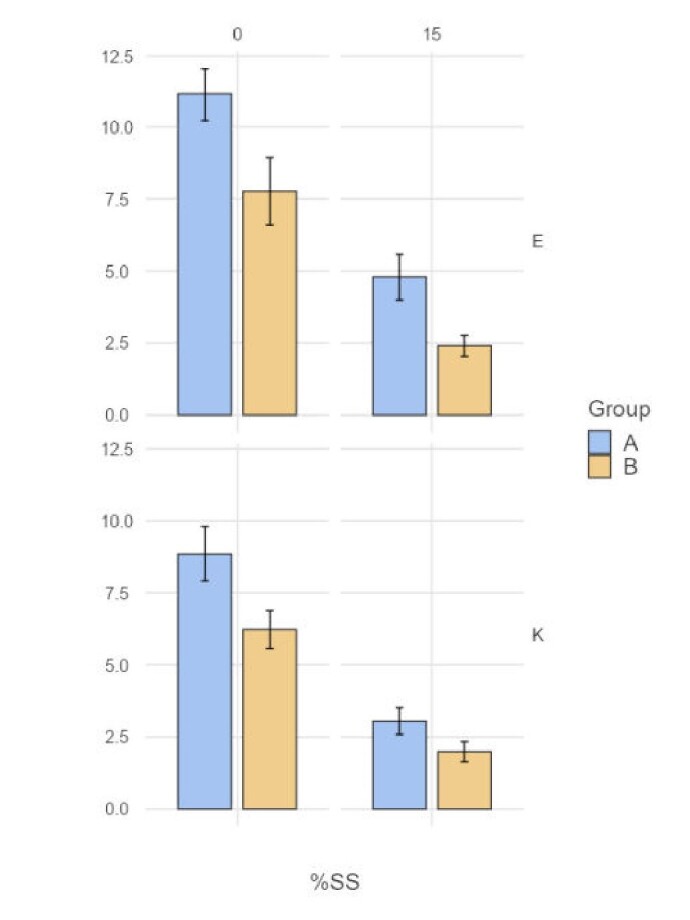
Pre (0) and post-intervention (15) %SS for participants in Group A and Group B. Abbreviations: E, English; K, Kannada, %SS, Percentage of stuttered syllables

To examine the significance of this effect, we conducted a linear mixed analysis (Table 4). The Linear Mixed Effects model (%SS ~ 1 + Group + Language + Timepoint + Group: Language + Group: Timepoint + Language: Timepoint + Group: Language: Timepoint+( 1 + Group + Language + Timepoint | Participant) showed a significant main effect of the Timepoint (F (1,14.9) = 69.58, p<0.001) and Language (F (1,46.26) = 13.91, p<0.001). That is, %SS showed a significant reduction from the baseline (Mean = 8.5; SE = 0.93) to the end of the intervention (Mean = 3.03; SE = 0.57), and the same was higher in English (Mean = 6.51; SE = 7.83) compared with Kannada (Mean = 5.02; SE = 0.67) (Figure 3). However, the main effect of Group (F (1,11.1) = 3.05, p=0.11) was not significant. Along with this, the interaction between the Group and Timepoint (p=0.36), Group and Language (p=0.19), Timepoint and Language (p=0.25), and all these three variables (p=0.68) did not reach statistical significance.

**Table 4:** Fixed effects parameters estimate

	95%CI							
Names	Effect	Estimate	SE	Lower	Upper	df	t	P value
(Intercept)	(Intercept)	5.731	0.690	4.379	7.083	11.6	8.3-9	<0.001*
Group	B-A	-2.456	1.379	-5.160	0.247	11.6	-1.781	0.101
Language	K-E	-1.482	0.438	-2.341	-0.623	32.8	-3.380	0.002
Time point	15-0	-5.528	0.413	-6.338	-4.718	163.6	-13.379	<0.001*
Group * Language	B-A * K-E	1.067	0.877	-0.652	2.785	32.8	1.217	0.232
Group * Timepoint	B-A * 15-0	1.096	0.826	0.523	2.716	163.6	1.327	0.186
Language * Timepoint	K-E *15-0	0.872	0.817	-0.729	2.472	161.8	1.068	0.287
Group *Language *Timepoint	B-A * Language *Time Point	0.635	1.633	-2.566	3.836	161.8	0.389	0.698

* p<0.05

**Caption:** CI, Confidence Interval; SE, Standard error; df, degrees of freedom; t, test statistic; A, Group A, B, Group B, K, Kannada language, E, English language

The percentage of syllables stuttered (%SS) may vary between participants even when their stuttering is categorized to the same degree (such as mild or moderate stuttering). Interclass correlation variation (ICC) was measured to examine the variability in the %SS between the subjects and was found to be 0.405.

Cronbach's alpha was used to assess the inter-rater and intra-rater reliability of the outcome measure (%SS). For intra-rater reliability, the first author reanalyzed 20% of the data six months apart. For inter-rater reliability, an independent assessor, blind to the study outcomes, assessed 20% of the data. Both inter-rater (α=0.98) and intra-rater reliability (α=0.95) were found to be high.

## DISCUSSION

The current study aimed to examine the motor and linguistic nature of stuttering by experimentally controlling the language of treatment in BWS. The left inferior frontal cortex was selected as the site for tDCS stimulation as this area is consistently shown to have abnormal activation patterns in individuals who stutter^(2-7)^. This area has also shown a reduction in stuttering post-stimulation using tDCS^(21,22)^. In the current study, stimulating this area with tDCS along with behavioral tasks yielded positive results in reducing stuttering dysfluencies.

Results from the analysis showed a reduction in stuttering dysfluencies was noted in both the languages spoken by bilinguals (English and Kannada) in both groups A (treated in dominant language) and B (treated in non-dominant language), regardless of the selection of language for treatment.

Stuttering is a speech-motor disorder that occurs due to neural deficits in the left motor areas^(7)^. The findings from this study support the notion that targeting left motor areas in the brain with current stimulation (tDCS) could contribute to improved speech fluency, regardless of the linguistic factors in play. The observed reduction in stuttering dysfluencies in both the treated and untreated language suggests a bi-directional generalization of the intervention effects, thus cementing the motoric nature of dysfluencies in stuttering.

Many linguistic factors have been reported to play a role in the generalization of treatment gains and in the differential manifestation of stuttering in bilinguals. The reported factors include language-related factors such as language proficiency or dominance^(13)^, linguistic similarity between the two languages^(12)^ that a bilingual speaks, and the structure of the languages. However, in the current experiment, these factors did not surface to play a role in cross-linguistic generalization of treatment gains from one language to another.

Cross-linguistic generalization in BWS is reported to occur in languages that are related in terms of belonging to the same language families or having a similar language structure^(12)^. However, the generalization observed in the current study cannot be explained with this factor as the languages spoken by the participants (i.e., English and Kannada) are neither related to each other in terms of their structure nor the terms of their language families^(23, 24)^. Kannada belongs to the family of Dravidian languages^(25)^, whereas English is an Indo-European language^(26)^. These languages are structurally and phonetically different from each other^(27)^. Hence, the structural or phonetic similarities may not be essential in the cross-linguistic generalization of treatment effect in stuttering, supporting the Vong et al.^(14)^ study, where generalization to untreated language was observed in linguistically and phonetically distinct languages (i.e., Mandarin and English).

Second, authors have speculated on the role of several factors, such as language proficiency or dominance, in cross-linguistic generalization of treatment gains^(13)^. It is assumed that even when individuals receive treatment in a non-dominant language, the stuttering levels may not show a comparable reduction as when a dominant language is treated. However, in the current study, the dominance of the participants was experimentally controlled, and the participants were randomized to receive treatment either in their dominant or non-dominant language. It was observed from the study findings that the treatment gains generalized to untreated language regardless of the dominance status of the treated language.

Yet another factor believed to play a role in cross-linguistic generalization in bilinguals is the practice of learned speech techniques in the untreated language^(12,13)^. Treatment of stuttering usually involves employing speech modification strategies to alter the natural characteristics of speech^(28)^, with participants being instructed to use these techniques consistently to maximize treatment outcomes. For BWS, adhering strictly to these instructions in only the language of treatment can become challenging, and these individuals may inadvertently apply the speech modification strategies in the untreated language beyond clinical settings. Consequently, it becomes difficult to assess cross-linguistic generalization as it remains unclear whether the participants used the treatment techniques solely in the treated language or in both the languages they speak^(12,13)^. In this current study, the behavioral techniques used included temporary fluency-enhancing conditions (choral speech and metronome speech)^(21)^, and the participants were not given any explicit instructions to use these techniques beyond clinical settings. The neural effects observed during these fluency-enhancing conditions are temporary and revert to pre-treatment levels once fluency-enhancing conditions are no longer applied. However, when combined with tDCS, these techniques have been shown to cause lasting changes in speech networks by neuromodulating the activity in the left IFC^(29,30)^. Similarly, in the current study, using these behavioral techniques, combined with tDCS, could have improved speech fluency in both languages of the individuals with stuttering.

The results obtained in this study find support from the results of neuroimaging data in PWS, which show motor-speech areas to be implicated in stuttering. Deficits in the left inferior frontal cortex, specifically in the SMA^(2,4,31)^, abnormal activation in left inferior frontal areas^(32)^, speech motor initiation, and execution have been identified to be aberrant in (PWS)^(1,33,34)^. The site of stimulation in this study was FC5 as per the 10-10 electrode positioning system^(35)^. Stimulating these areas showing a reduction in stuttering dysfluencies suggests tDCS could have led to the normalization of aberrant activation patterns in this area as tDCS is shown to enhance synaptic plasticity when applied in combination with a potentiating neuronal activity^(36,37)^. Combining tDCS along with a specific behavioral task is shown to enhance the tDCS effect and show improvement in behavioral tasks along with neural gains^(38)^ by supporting efficient inferior frontal cortex function during speech, leading to improvement in stuttering^(22)^.

Overall, cross-linguistic generalization from the dominant to non-dominant language in Group A and Non-Dominant to Dominant in Group B render limited evidence for the role of linguistic factors playing a role in stuttering. This study is not without limitations. The stuttering severity of the participants varied, making it a heterogeneous group. Language factors were controlled at baseline for randomization; however, no measure was taken to randomize participants based on their stuttering severity.

## CONCLUSION

The core nature of dysfluencies in stuttering remains whimsical. Researchers suggest stuttering is a pure speech motor disorder; however, others suspect linguistic factors play a role in the occurrence of dysfluencies. In the current study, bilinguals with stuttering were randomized to receive stuttering intervention using behavioral treatment (in dominant or non-dominant language) and transcranial direct current stimulation. Several factors suspected to play a role in cross-linguistic generalization, such as language-related factors and treatment practice effect, were controlled in the study. The findings from the study show that cross-linguistic generalization occurred even when linguistic factors were regulated, suggesting the possibility of motor factors playing a role in stuttering disfluencies.

## References

[1] Watkins KE, Smith SM, Davis S, Howell P (2008). Structural and functional abnormalities of the motor system in developmental stuttering. Brain.

[2] Connally EL, Ward D, Howell P, Watkins KE (2014). Disrupted white matter in language and motor tracts in developmental stuttering. Brain Lang.

[3] Busan P (2020). Developmental stuttering and the role of the supplementary motor cortex. J Fluency Disord.

[4] Budde KS, Barron DS, Fox PT (2014). Stuttering, induced fluency, and natural fluency: a hierarchical series of activation likelihood estimation meta-analyses. Brain Lang.

[5] Neumann K, Preibisch C, Euler HA, Von Gudenberg AW, Lanfermann H, Gall V (2005). Cortical plasticity associated with stuttering therapy. J Fluency Disord.

[6] Etchell AC, Civier O, Ballard KJ, Sowman PF (2018). A systematic literature review of neuroimaging research on developmental stuttering between 1995 and 2016. J Fluency Disord.

[7] Belyk M, Kraft SJ, Brown S (2017). Stuttering as a trait or a state revisited: motor system involvement in persistent developmental stuttering (vol 41, pg 275, 2015). Eur J Neurosci.

[8] Dworzynski K, Howell P (2004). Predicting stuttering from phonetic complexity in German. J Fluency Disord.

[9] Howell P (2004). Assessment of some contemporary theories of stuttering that apply to spontaneous speech. Contemp Issues Commun Sci Disord.

[10] Yang Y, Jia F, Fox PT, Siok WT, Tan LH (2019). Abnormal neural response to phonological working memory demands in persistent developmental stuttering. Hum Brain Mapp.

[11] Hakim HB, Ratner NB (2004). Nonword repetition abilities of children who stutter: an exploratory study. J Fluency Disord.

[12] Priyanka K, Maruthy S (2019). Cross-linguistic generalization of fluency to untreated language in bilingual adults who stutter. Journal of Indian Speech Language & Hearing Association..

[13] Lim VP, Lincoln M, Onslow M, Chan YH (2015). English-only treatment of bilingual speakers who stutter: Generalization of treatment effects from English to Mandarin. Int J Speech Lang Pathol.

[14] Vong E, Wilson L, Lincoln M (2016). The Lidcombe Program of early stuttering intervention for Malaysian families: four case studies. J Fluency Disord.

[15] Ardila A, Ramos E, Barrocas R (2011). Patterns of stuttering in a Spanish/English bilingual: a case report. Clin Linguist Phon.

[16] Lim VP, Lincoln M, Chan YH, Onslow M (2008). Stuttering in English–Mandarin bilingual speakers: the influence of language dominance on stuttering severity. Journal of Speech, Language, and Hearing Research.

[17] Schäfer M, Robb MP (2012). Stuttering characteristics of German–English bilingual speakers. Clin Linguist Phon.

[18] Maruthy S, Raj N, Geetha MP, Priya CS (2015). Disfluency characteristics of Kannada–English bilingual adults who stutter. J Commun Disord.

[19] Riley G, Bakker K. (2009). SSI-4: stuttering severity instrument.

[20] Jamil A, Batsikadze G, Kuo HI, Meesen RL, Dechent P, Paulus W (2020). Current intensity‐and polarity‐specific online and aftereffects of transcranial direct current stimulation: an fMRI study. Hum Brain Mapp.

[21] Chesters J, Möttönen R, Watkins KE (2018). Transcranial direct current stimulation over left inferior frontal cortex improves speech fluency in adults who stutter. Brain.

[22] Chesters J, Watkins KE, Möttönen R (2017). Investigating the feasibility of using transcranial direct current stimulation to enhance fluency in people who stutter. Brain Lang.

[23] Maruthy S, Venugopal S, Parakh P (2017). Speech rhythm in Kannada speaking adults who stutter. Int J Speech Lang Pathol.

[24] Ononiwu CA (2010). The impact of syllable structure complexity on stuttering frequency for bilinguals and multilinguals who stutter.

[25] Krishnamurti B. (2003). The Dravidian languages.

[26] Makarova EA, Polyakov VN (2015). The origin of the article in Indo-European Languages of Western Europe. Mediterr J Soc Sci.

[27] Grolman MB, Biktagirova ZA, Kasimov OH (2021). Phonetic peculiarities of the English language in India. Int J Soc Cult Lang..

[28] Blomgren M, Roy N, Callister T, Merrill RM (2005). Intensive stuttering modification therapy: a Multidimensional. J Speech Lang Hear Res.

[29] Chesters J, Möttönen R, Watkins KE (2021). Neural changes after training with transcranial direct current stimulation to increase speech fluency in adults who stutter. OSF Preprints.

[30] Busan P, Moret B, Masina F, Del Ben G, Campana G (2021). Speech fluency improvement in developmental stuttering using non-invasive brain stimulation: insights from available evidence. Front Hum Neurosci.

[31] Brown S, Ingham RJ, Ingham JC, Laird AR, Fox PT (2005). Stuttered and fluent speech production: an ALE meta‐analysis of functional neuroimaging studies. Hum Brain Mapp.

[32] Chang SE (2011). Using brain imaging to unravel the mysteries of stuttering. Cerebrum.

[33] Chang SE, Zhu DC, Choo AL, Angstadt M (2015). White matter neuroanatomical differences in young children who stutter. Brain.

[34] Neef NE, Anwander A, Friederici AD (2015). The neurobiological grounding of persistent stuttering: from structure to function. Curr Neurol Neurosci Rep.

[35] Acharya JN, Acharya VJ (2019). Overview of EEG montages and principles of localization. J Clin Neurophysiol.

[36] Buchwald A, Khosa N, Rimikis S, Duncan ES (2020). Behavioral and neurological effects of tDCS on speech motor recovery: A single-subject intervention study. Brain Lang.

[37] Kronberg G, Rahman A, Sharma M, Bikson M, Parra LC (2020). Direct current stimulation boosts hebbian plasticity in vitro. Brain Stimul.

[38] Chan MM, Han YM (2022). The functional brain networks activated by music listening: a neuroimaging meta-analysis and implications for treatment. Neuropsychology.

